# Financial impact of COVID-19 on TB patients in India

**DOI:** 10.5588/ijtld.21.0658

**Published:** 2022-03-01

**Authors:** S. Chatterjee, P. Das, A. Vassall

**Affiliations:** 1 Research Department, George Institute for Global Health, New Delhi, India; 2 Department of Medicine, University of New South Wales, Kensington, NSW, Australia; 3 Prasanna School of Public Health, Manipal Academy of Higher Education, Manipal, Karnataka, India; 4 Department of Global Health and Development, London School of Hygiene & Tropical Medicine, London, UK

Dear Editor,

The nationwide response to curb the spread of the COVID-19 infection in India included a strict lockdown from 25 March 2020 until the end of May, with several restrictions remaining in place for 2 further months. Studies have reported severe economic crisis and serious health service disruptions during this lockdown.^1^–^6^ TB services were also disrupted, with a significant decline in TB notifications during the strict lockdown period, which is estimated to lead to an increase of 182,000 TB cases and 83,600 deaths between 2020 and 2025.^7^,^8^ However, there is only limited empirical evidence on the economic impact of COVID-19 on other diseases. We report the financial impact of the pandemic on TB patients in India along with TB service use patterns before and during lockdown.

As part of an ongoing study, we collect primary data on patient and household costs from four states to estimate TB-related out-of-pocket expenses and catastrophic expenditure in India. Here, we report on the financial impact of COVID-19 as income loss for the families of TB patients during lockdown based on follow-up interviews in two states. We examined monthly household income of the sampled TB patients just before lockdown (February 2020) and during lockdown (April and May 2020) to understand the impact on household income. Monthly household income during lockdown and during the interview period (July and August 2020) were subtracted from monthly household income in February 2020 to estimate any income loss. To ascertain whether the pattern of TB drug collection had changed as a result of the lockdown, patients were asked about the frequency of visits to get drugs, amount of time spent travelling and collecting drugs, and travel and other expenses related to drug collection for two time periods – before lockdown and during lockdown. The time costs for the patient, household member and accompanying person were calculated by multiplying the total number of hours spent on the activity by the hourly wage. To determine if any TB medication was discontinued during lockdown, patients were asked whether they missed their medicine because of the lockdown. Household income loss was presented in 2020 US dollars (US$) (1 US$ = Indian rupee 74.132). The study covered 202 TB patients from the general population, 379 patients from tea garden areas and 69 patients from urban slum dwellers in the two states.

The impact of COVID-19 on household incomes of the sampled patients are shown in the accompanying Figure. Before lockdown, 6% of TB patient families in the general population had zero household income, which increased to 54% during the strict lockdown. The proportion of households in different income brackets decreased significantly during lockdown compared to the pre-lockdown period, indicating a high rate of unemployment during the lockdown period. Similar trends were observed for the other two groups (Figure). If the income loss estimated in our study is representative of households with TB patients across India, the total income loss in 2 months would amount to US$460 million. This loss was gradually reversed over the next 3 months (June–August 2020): in that period, income loss was estimated at US$116 million. In general, the number of visits for drug collection during lockdown was lower for all groups and consequently, the total number of hours spent and time costs were lower than the pre-lockdown period. However, for patients in tea garden areas, total expenses related to drug collection (travel and other expenses on food or payment related to drug collection) was higher during lockdown. This is likely to be due to the remote locations and lack of public transportation. About 7% of the patients in the general population and 4% of those in tea garden areas discontinued their TB medication during lockdown; the average number of days of medicine discontinuation were respectively 10 and 8.5. The relatively lower proportion of discontinuation in tea garden areas was probably because many tea gardens patients continued to be administered DOT even during lockdown. Other reasons for medicine discontinuation in both groups of patients included the lack of TB medicines or the patient’s inability to travel due to strict travel restrictions.

**Figure:**
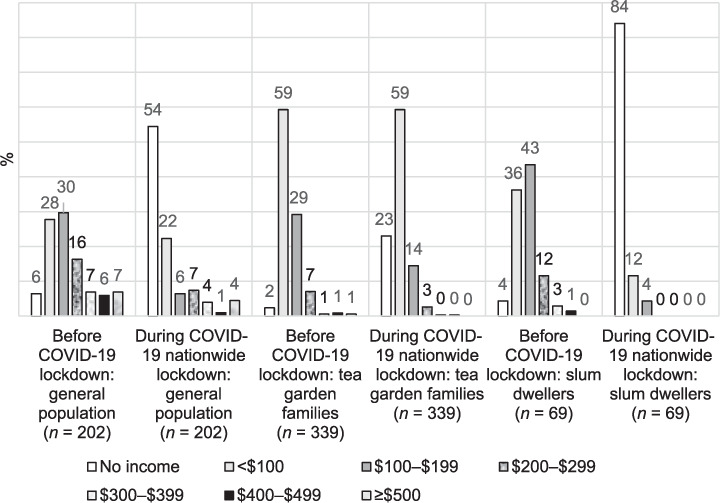
Percentage of households in different income groups before COVID-19 lockdown (February 2020) and during the COVID-19 nationwide lockdown, April–May 2020, India.

The COVID-19 pandemic hit India at a time when India’s gross domestic product was slowing down, and unemployment was rising because of poor economic performance in recent years.^9^ The pandemic affected all sectors of society; however, workers employed in the informal sector (which contributes about 91% of total employment in India) was the worst affected.^1^,^3^,^9^ Our results corroborate this finding, as the majority of the sampled TB patients who were employed before the pandemic were involved in the informal sector. The proportion was higher for patients among urban slum dwellers and thus, suffered the greatest financial impact on household income (Figure).

Studies have reported reduced food consumption during the pandemic.^1^,^3^,^6^ In one study, 44% of 3,466 households interviewed reported that they had borrowed money to meet their daily requirements and 14% of households occasionally did not have enough food because of the financial crisis.^1^ Our study findings also indicate that income loss for the households and poor restoration of income after lockdown will have had a serious impact on the nutrition of the TB patients and families. It is well-recognised that TB disproportionately affects poor people and there is a bidirectional relationship between undernutrition and TB.^10^–^13^ TB treatment interruption during the lockdown is also a major concern as it is related to poor treatment outcomes and the possibility of acquiring multidrug-resistant TB in future.^14^,^15^ Therefore, not only will a lower rate of diagnosis during lockdown impact the number of TB cases, but also the discontinuation of medication will impose an additional burden in the future.

The Government of India provided some relief measures with additional rations (grains and pulses) and additional cash transfers during the lockdown period; however, these were modest and there were disparities in distribution across states and between rural and urban areas.^1^ Generally, a much lower number of urban households received government assistance. It is therefore likely that TB patients living in urban slum areas – the worst affected group during the pandemic – did not receive sufficient support from the government. In future, government safety net measures must be designed to provide support for the most vulnerable groups (e.g., informal workers) and to protect people with comorbidities, including people living with TB.

## References

[1] National Council of Applied Economic Research (2020). Round 3 of the Delhi NCR Coronavirus Telephone Survey. https://www.ncaer.org/image/userfiles/file/NDIC-TEL/Round-3/NCAER_DCVTS_Round-III-Press_Release.pdf.

[2] Chandra Shekar K, Mansoor K (2020). COVID-19: lockdown impact on informal sector in India. https://practiceconnect.azimpremjiuniversity.edu.in/covid-19-lockdown-impact-on-informal-sector-in-India/.

[3] Dalberg (2020). Minimizing the impact of the pandemic on India’s most vulnerable populations. https://dalberg.com/our-ideas/minimizing-the-impact-of-the-pandemic-on-indias-most-vulnerable-populations/.

[4] World Bank (2021). Economic effects of COVID-19: rapid surveys of rural households in India. https://pubdocs.worldbank.org/en/645971613651626018/Economic-Effects-of-COVID19-Rapid-Rural-Surveys.pdf.

[5] Nguyen PH (2021). COVID-19 disrupted provision and utilization of health and nutrition services in Uttar Pradesh, India: insights from service providers, household phone surveys, and administrative data. J Nutr.

[6] Singh K (2021). Health, psychosocial, and economic impacts of the COVID-19 pandemic on people with chronic conditions in India: a mixed methods study. BMC Public Health.

[7] Cilloni L (2020). The potential impact of the COVID-19 pandemic on the tuberculosis epidemic a modelling analysis. EClinicalMedicine.

[8] Stop TB Partnership, Imperial College, Avenir Health, Johns Hopkins University, USAID (2020). The potential impact of the Covid-19 response on tuberculosis in high-burden countries: a modelling analysis. http://www.stoptb.org/assets/documents/news/Modeling%20Report_1%20May%202020_FINAL.pdf.

[9] Dev S, Sengupta R (2020). Covid-19: impact on the Indian economy. Mumbai Working Papers 2020-013.

[10] Gupta D (2004). Role of socio-economic factors in tuberculosis prevalence. Indian J Tuberc.

[11] Bhargava A (2016). Undernutrition, nutritionally acquired immunodeficiency, and tuberculosis control. BMJ.

[12] Oxlade O, Murray M (2012). Tuberculosis and poverty: why are the poor at greater risk in India. PLoS One.

[13] Padmapriyadarsini C (2016). Undernutrition & tuberculosis in India: situation analysis & the way forward. Indian J Med Res.

[14] Babiarz KS, Suen S, Goldhaber-Fiebert JD (2014). Tuberculosis treatment discontinuation and symptom persistence: an observational study of Bihar, India’s public care system covering >100,000,000 inhabitants. BMC Public Health.

[15] Tola HH (2019). Intermittent treatment interruption and its effect on multidrug resistant tuberculosis treatment outcome in Ethiopia. Sci Rep.

